# Instantiating the onEEGwaveLAD Framework for Real-Time Muscle Artefact Identification and Mitigation in EEG Signals

**DOI:** 10.3390/s25165018

**Published:** 2025-08-13

**Authors:** Luca Longo, Richard Reilly

**Affiliations:** 1 The Artificial Intelligence and Cognitive Load Research Lab, Centre of Explainable Artificial Intelligence, Technological University Dublin, D07 EWV4 Dublin, Ireland; 2 Trinity Centre for Biomedical Engineering, Trinity College Dublin, D02 X9W9 Dublin, Ireland

**Keywords:** electroencephalography, muscle artefacts, real-time denoiser, discrete wavelet transform, Isolation Forest, machine learning, signal processing and restoration, sliding moving buffer

## Abstract

While electroencephalography is extremely useful for studying brain activity, EEG data is always contaminated by a wide range of artefacts. Many techniques exist to identify and remove such artefacts, primarily offline, with and without human supervision and intervention. This research presents a novel, fully automated online wavelet-based learning adaptive denoiser for artefact identification and mitigation in EEG signals. It contributes to knowledge by offering a framework that can be instantiated with artefact-specific and context-dependent parameters. In detail, this framework is instantiated for block online muscle artefact identification and mitigation. It is based on the discrete wavelet transformation (DWT) for time–frequency enrichment and the Isolation Forest algorithm for linearly learning data characteristics and identifying anomalous activity in a sliding moving buffer. It is built upon a denoising strategy that operates in the domain of DWT coefficients before reverting characteristics to the time domain. The findings demonstrate that such instantiation is promising in its goal of successfully identifying myogenic muscle movements and transforming them into cleaner EEG signals. They also emphasise the difficulties in tackling the known problem of the cone of influence associated with wavelet transformation and the tradeoff between the length of consecutive EEG windows and the problem’s real-time nature.

## 1. Introduction

Artefacts often hinder the utility of electroencephalography for studying the human brain in recorded signals at the scalp level. One of these artefacts is represented by myogenic potentials generated by muscle movements, which are usually significantly stronger than genuine neural signals. Several techniques have been developed and proposed for identifying and reducing these types of artefacts in EEG recordings. Examples include those based on independent component analysis (ICA) and canonical correlation analysis (CCA) [1], which often require human supervision and multi-channel data for effective decomposition. Also, these require a significant amount of data to converge, and are often used offline, taking advantage of the entirety of recorded EEG signals. Other denoising methods are fully unsupervised, such as Fully Automated Statistical Thresholding for EEG Artefact Rejection (FASTER) [2], but again they require multi-channel data to exhibit reasonable capabilities in identifying and mitigating muscular activity. While fully automated, these types of methods are not suitable for real-time applications.

For these reasons, a new class of methods is being developed to design and implement online EEG artefact denoisers. While applicable in brain–computer interfaces, they are not simple to devise and develop. One reason is that they operate on blocks of EEG data that might be too small to identify artefacts. Additionally, they cannot use an entire EEG recording, as their manipulation must be continuously performed in pseudo-real time. Eventually, most of them require multi-channel data, calibration phases that are often supervised and parameterised, and they do not work in single-channel settings. To tackle the above issues in the discipline of neuroscience, particularly in the fields of electroencephalography and signal processing, this study builds on the *onEEGwaveLAD* framework [3], a fully automated online EEG wavelet-based learning adaptive denoiser for artefact identification and mitigation that work in single-channel settings and do not require supervision or heavy calibration phases. This framework can be instantiated with different artefact-specific and context-dependent parameters. This study specifically contributes to the body of knowledge by instantiating *onEEGwaveLAD* to identify and reduce muscle artefacts.

The remainder of this manuscript is devoted to the presentation and discussion of related work in Section 2. This includes describing muscle artefacts and their characteristics in the time, spatial, and frequency domains. It also expands the description of techniques for identifying and reducing such artefacts, emphasising issues in online applications. Section 3 formally introduces the *onEEGwaveLAD* framework and all the layers it is built upon. Section 4 introduces the design and methods for instantiating *onEEGwaveLAD* for the particular problem of muscle artefact identification and reduction, along with the research hypothesis. This is followed by presentation of the findings with an in-depth discussion in Section 5 and a concluding part highlighting future areas of improvement for *onEEGwaveLAD* and future experimental work in Section 6.

## 2. Related Work

Body and muscle movements generate unavoidable myogenic potentials with amplitudes orders of magnitude higher than EEG signals, essentially covering them. The higher such movements and the activation of a group of muscles, the stronger such amplitudes, leading to high-frequency oscillations. These can be captured based on duration, spatial morphology, and frequency of occurrence. These oscillations are shorter than those typically generated by the brain and are prominent above 20 Hz. They can cover the beta band, reach up to 300 Hz [4], and are not fully visible in the delta, theta, and alpha bands. However, myogenic potentials generated by muscle movements cover a broad spectrum that impacts all the EEG frequencies [1]. The forehead, jaw, and, in general, the temporalis and lighter-power frontalis of the head are examples of muscles that contribute to such potentials. The shoulder or the neck can also generate muscle tension and produce persistent noise that can reach the lower electrodes. Similarly, teeth clenching can generate significant artefacts that can spatially propagate to the whole scalp. Many techniques for reducing body motions and muscle artefacts have been proposed [1,5]. By employing the categorisation described in [6], these can generally be divided into ‘manual’, ‘offline’, ‘block-online’, and ‘online’. In manual methods, the entire EEG recording is fully available and exploited offline, as seen in source decomposition methods, including independent component analysis (ICA) or canonical correlation analysis (CCA) [5,7]. Here, EEG data are decomposed into components, and those containing muscle artefacts, as visually assessed, are either rejected or manually manipulated. Offline methods are also based on the complete EEG recording, but they have a degree of automation that does not require human supervision [2,8,9]. Block-online methods do not exploit the whole EEG recording but only data segments or blocks, usually on the order of seconds; thus, human supervision is not feasible. These are often based on wavelet decomposition, such as FORCe [10], which uses segments that are 500 ms long. Online methods are very similar to the previous ones, but EEG data is processed almost instantaneously. Although theoretically capable of working on a sample-by-sample basis, they also operate on buffered segments in practice. However, these are relatively smaller (10 to 50 ms), and are thus often referred to as online or real-time methods [11].

Online denoisers are the most complex, as EEG data must be collected and processed in almost real time. Processing typically involves various signal transformations into the time–frequency domain, such as Fourier or wavelet transformations. It also entails automatic artefact identification in this domain, and automatic artefact corrections, such as manipulating the wavelet coefficients, and their reconstruction in the time domain [11]. Artefact reduction includes removing or attenuating wavelet coefficients using a thresholding mechanism [12]. A standard approach utilises the universal threshold, accounting for all wavelet coefficients and their standard deviation [13]. Usually, thresholds can be established by visually inspecting the wavelet coefficients. However, this is complex in online applications because such estimation must be performed in real-time, as EEG noise characteristics may vary dynamically. Subsequently, artefact reduction is usually implemented by applying a hard or soft thresholding function to the wavelet coefficients based on a specific threshold [14]. Threshold values can be generated by considering all the wavelet coefficients for all the DWT decomposition levels or separately for each of them [15], where stationarity is not guaranteed [16]. Recording, transforming EEG signals into the time–frequency domain, identifying artefacts in the signals, denoising them, and generating an artefact-free signal continuously over time, segment by segment, are challenges for developing an online denoiser.

## 3. The *onEEGwaveLAD* Framework

The *onEEGwaveLAD* [3] is an 8-tuple:

$$<RTW_{L},S_{r},MW,IF_{S},IF_{t},B_{s},T_{a},E_{s}>$$

where

- $RTW_{L}$ is the *-time EEG window length*, in milliseconds of an EEG segment;
- $S_{r}$ is the *sampling rate*, the number of points of an EEG segment, for dealing with the granularity of denoising;
- MW is the *mother wavelet*, a function used for decomposing an EEG segment, employing the DWT decomposition scheme;
- $IF_{S}$ is the *IF sub-sampling size*, the number of randomly sampled observations used to train each Extended Isolation Forest tree;
- $IF_{t}$ is the *number of IF trees* for the Extended Isolation Forest algorithm to use for learning;
- $B_{s}$ is the *buffer capacity*, the amount of EEG windows composing the sliding buffer for storing the past EEG signal’s behaviour in the current recorded window;
- $T_{a}$ is the *anomaly threshold*, a scalar for deeming a vector of *n*-dimensions (the decomposition scales) as an outlier given its anomaly score, computed by querying the Extended Isolation Forest model;
- $E_{s}$ is the *expansion step*, the time locations to consider around each anomalous vector that must be denoised.

### 3.1. Window Length and Sampling Rate

The $RTW_{L}$ is the first parameter of the *onEEGwaveLAD* framework; it is grounded on a sliding window technique, whereby consecutive windows of EEG data of length $RTW_{L}$ are individually collected and processed sequentially, one by one. The term ‘real-time’ refers to the specific multi-layer nature of the framework, a system that receives data, processes it, and returns results sufficiently quickly to perform meaningful actions at a given time, in this case, denoising EEG data [17,18]. The value for this parameter depends on factors such as the type of artefacts that need to be identified and mitigated. While muscle movements are transient and fast in the time domain, covering a broad range of frequencies (20–300 Hz) [4], ocular artefacts are slower, last longer (200 ms to 700 ms), and cover a smaller range of frequencies (4–20 Hz) [19]. Therefore, the window length for ocular artefacts should be approximately 700 ms, while it can be smaller for muscle artefacts. Intuitively, the larger the window $RTW_{L}$, the better it is for artefact identification, but the later the post-processing, which limits the real-time effect. Such a tradeoff is artefact-specific and context-dependent. $S_{r}$ refers to the sampling rate; that is, how many points are collected in one second: the higher the rate, the higher the quality and effectiveness of the post-processing. According to Nyquist’s theorem, a periodic signal must be sampled at over twice the highest frequency component of interest.

### 3.2. Single-Channel EEG Decomposition

MW is the third parameter of *onEEGwaveLAD*; it implements single-channel discrete wavelet transform (DWT). Such a transformation decomposes an EEG signal into an orthogonal basis obtainable by dilating and time-translating the mother wavelet (MW) with a function Ψ(t) and a scaling function Φ(t), returning a set of DWT coefficients. *onEEGwaveLAD* employs the pyramidal multiresolution scheme of Mallat [20,21] for efficiency purposes, whereby a signal x(t) of length *n* is decomposed into time–frequency components to a level m∈M, via a hierarchical set of sub-band filters. Such a coding scheme is chosen over the continuous wavelet transform (CWT) due to its efficiency, having a computational complexity of O(|M|∗n) [20], which is essential for real-time decomposition. Formally,

(1) $$x\left(t\right)=\sum_{n}C_{M,n}\Phi M,n\left(t\right)+\sum_{m=1}^{M}\sum_{n}d_{m,n}\Psi m,n\left(t\right)$$

with

(2) $$\Psi m,n\left(t\right)=2^{-m/2}\Psi \left(2^{-m}t-n\right)$$

(3) $$\Phi m,n\left(t\right)=2^{-m/2}\Phi \left(2^{-m}t-n\right)$$

where $C_{m,n}=<x,\Phi m,n>$ and $d_{m,n}=<x,\Psi m,n>$ are, respectively, the approximation and detail coefficients at level *m*. The scalar product is defined with $<f,g>=\sum_{t\in Z}f\left(t\right)g\left(t\right)$. The selection of the mother wavelet MT (Ψ(t)) depends on the domain of application and the characteristics of the underlying signal [22,23,24]. The output of the DWT transformation of a univariate *x* EEG signal is a set of approximations and detailed coefficients for each decomposition level in *M*. Due to the recursive nature of the sub-band scheme, each decomposition level contains half of the coefficients of the previous level. The decomposition scheme terminates when no further splits can be applied. Readers are referred to [20,21] for the details of Mallat’s decomposition scheme. Generally speaking, DWT offers poor resolution in the time domain for low frequencies and reasonable resolution for high frequencies, making it an effective data reduction strategy. It is important to note that the number of decomposition levels depends on the length of the original signal. For efficiency purposes, and due to its recursive sub-sampling by 2, the window length $RTW_{L}$ must be a power of 2, or at least a multiple of a power of 2.

#### 3.2.1. The Cone of Influence and Edge Effects

A problem with DWT is the generation of edge effects, a phenomenon often referred to as the cone of influence [25,26]. In particular, mother wavelets applied near the edges of an EEG window $RTW_{L}$ inevitably extend their domain outside. Thus, DWT coefficients close to these edges are hampered and should be carefully interpreted and applied. While various techniques have been designed to compensate for this effect, including the symmetrical reflection of the signal at the border or its periodic extension, no consensus exists [27,28]. The strategy used in the *onEEGwaveLAD* framework is based on the fact that, at each instant point in time, past recorded EEG windows can be exploited, but not the future ones. This means that the problem of edge effects on the left border of a current EEG window $w_{c}$ can be reasonably resolved by concatenating the current window to the previous one $w_{c-1}$ and then passing such concatenation ($conc=<w_{c-1},w_{c}>$) to the DWT decomposition. In this way, the left border of $w_{c}$ is now in the middle of conc, so it is no longer affected by edge effects. Unfortunately, this does not work for the right border of $w_{c}$ since no window follows it, and a smooth-padding mode strategy is adopted, whereby the signal of $w_{c}$ is extended according to the first derivatives calculated on the edges (straight line) [29]. These operations introduce additional DWT coefficients on the left and right sides of $w_{c}$, which will eventually be trimmed before the inverse composition to the time domain.

#### 3.2.2. Asymmetry in DWT Decomposition and Scaleogram Formation

One problem associated with the pyramidal scheme is the varying number of DWT coefficients generated at each decomposition level, which makes visual inspection and understanding rather cumbersome. To solve this, the notion of a ‘scaleogram’ is considered, a visualisation similar to a spectrogram but specifically suitable for DWT coefficients. This proposes that the asymmetry in the number of DWT coefficients at various levels can be resolved by replicating those at lower levels by twice the number of those at the level above. This effectively forms a matrix of coefficients that can now be visually depicted, facilitating the interpretation of the energy over time at different frequencies. Technically, the DWT coefficients at lower levels (high frequencies) are generally weaker in magnitude than those at higher levels (lower frequencies), which may potentially have different importance if used in subsequent computations. *onEEGwaveLAD* proposes a normalisation strategy at the different decomposition levels (the scales) to tackle this. This divides each coefficient *d* at level *m* by $2^{m}$, with *m* starting at 1. Formally,

(4) $$d_{norm}=\frac{d}{2^{m}}$$

with $d_{norm}$ the normalised DWT coefficient at level *m*. The normalised matrix is of shape n×m, (with *n* the normalised DWT coefficients, and *m* the decomposition levels). Each slice of the normalised DWT matrix (scaleogram) is an *n*-dimensional vector which describes the signal at a given point in time.

### 3.3. Artefact Identification via a Moving Buffer and the Isolation Forest Anomaly Detector

An essential assumption of *onEEGwaveLAD* is that the particular vectors that describe portions of artefacts in the underlying EEG signal are rare and uncommon. Thus, their identification can be tackled with an anomaly detection approach [30]. However, while many of these approaches exist, often with linear time complexity in detection, they are grounded in training procedures that usually have quadratic complexities in time and memory [30]. These are not suitable for real-time anomaly detection applications. To account for this, *onEEGwaveLAD* employs the Isolation Forest (iForest) algorithm, an approach with linear training-time complexity and a limited memory consumption requirement [31]. This is a model-free and fully unsupervised approach because it does not require probabilities or statistics, including density estimation or class distribution statistics, and it operates effectively without ground truth on n-dimensional data. The assumption is that anomalies are specific instances of a dataset with characteristics in n-dimensional space that are significantly different from the rest and can be easily identified.

$IF_{s}$ is a parameter of *onEEGwaveLAD* that is employed in a sub-sampling procedure of the Isolation Forest algorithm for solving the swamping issues, which means the proximity of regular points to the anomalous one/s, and the masking problem when many anomalies exist [32]. Technically, given a dataset $X=x_{1},\ldots ,x_{c}$, with each instance of dimension *c*, iForest sub-samples a random portion of data $X^{\prime }<X$ to construct a binary tree (Isolation Tree, iTree). Such a tree goes into a branching mechanism by picking a random dimension $rd_{i}$, with *i* in 1,2,…,c, and a random split value sv, within the range of $rd_{i}$ ($sv\in [min\left(rd_{i}\right),max\left(rd_{i}\right)]$). If the dimension $rd_{i}$ of a particular data instance has a value smaller than sv, then such an instance is assigned to the left branch of the iTree, otherwise to the right. Branching is recursive for each instance until a single point is isolated or a predetermined depth limit is reached. Formally, an iTree is a specific structure where each node *T* is either a leaf, an external node with no child, or an internal node with precisely two children nodes ($T_{l}$, $T_{r}$), and a condition defined by a dimension $rd_{i}$, and a split value sv, such that $rd_{i}<sv$ determines the traversal of a data point to the left branch $t_{l}$, or the right branch $T_{r}$. An iTree is fully grown when each point in the dataset *X* is isolated at one of the leaf nodes. Each data point $x_{i}\in X$ has a specific path of length $h(x_{i})$, which equates to the number of edges $x_{i}$ traverses from the root to a leaf in the tree. In line with the previous assumption, an anomaly is a data point with a smaller path length that can be isolated easily.

$IF_{t}$ is a parameter of the *onEEGwaveLAD* pipeline; it is the number of times the aforementioned recursive training phase is repeated. This leads to an ensemble of iTrees, an Isolation Forest (iF) on which binary search can be performed to identify anomalies. Analogously to the strategy of the Binary Search Trees (BST) algorithm [33], if the searching mechanism terminates at a leaf node on an iTree, then it is unsuccessful. An unsuccessful search in BST equates to estimating the average path length h(x) of all leaf nodes, which is equivalent. Formally,

(5) $$a(|X^{\prime }\left|\right)=\left\{\begin{array}{cc} 2H(|X^{\prime }\left|-1\right)-\frac{2(|X^{\prime }|-1)}{\left|Y\right|} & \mathrm{for}\;|X^{\prime }|>2\\ 1 & \mathrm{for}\;|X^{\prime }|=2\\ 0 & \mathrm{otherwise} \end{array}\right.$$

with *Y* the testing data and |Y| the cardinality; $|X^{\prime }|$ is the size of the random portion of data $X^{\prime }$; *H* is the harmonic number, estimable by H(i)=ln(i)+γ (with γ=0.5772156649 the Euler–Mascheroni constant). $a(|X^{\prime }|)$ can be seen as the average of h(x) given $|X^{\prime }|$, and can be used to normalise h(x) for estimating a specific data instance’s anomaly score x∈X:

(6) $$s(x,a(|X^{\prime }\left|)\right)=2^{\frac{-E(h(x))}{a(a(|X^{\prime }\left|)\right)}}$$

where E(h(x)) is the average value of h(x) from the collection of iTrees (forest). For any instance x∈X, if *s* is smaller than 0.5, then *x* is likely to be a common value; otherwise, if *s* is close to 1, the instance *x* is likely anomalous. When the forest is entirely constructed, each instance of a test set $Y=y_{1},\ldots ,y_{c}$ is passed to each of its iTrees, and an anomaly score is computed. The *onEEGwaveLAD* pipeline utilises the Extended Isolation Forest algorithm [34], a specialised version designed to address the bias issues of the original algorithm resulting from the tree branching procedure.

The $B_{s}$ parameter of *onEEGwaveLAD* is the size of the sliding buffer. While it is theoretically possible to consider all the vectors of each normalised scaleogram up to a point in time, training an Isolation Forest requires a linear increment in time complexity, proportional to the number of recorder EEG windows, making it a bottleneck for the practical development of a real-time denoiser. Thus, the buffer $B_{s}$ is of fixed capacity *s* and aims at containing the *n*-dimensional vectors of the normalised scaleograms of the *s* recorded EEG windows before the one recorded in real time. Such a buffer gives *onEEGwaveLAD* its adaptivity property because it is intrinsically adjustable to the changing recording environment, inherent in EEG measurements due to the slow amplitude drifts in each EEG channel due to the drying conductive gel or the decrease in adherence of the electrodes to the scalp. This introduces a second assumption: genuine neural signals are present in most parts of such a buffer, with a minimal portion of artefacts. If this is not met, detecting anomalies will be significantly hampered if the data mainly contains artefacts. The investigation of this assumption is left for future work.

$t_{a}$ is a parameter of *onEEGwaveLAD* used after the Isolation Forest algorithm is trained and queried with the *n*-dimensional vectors in the moving buffer, producing a list of anomaly scores for each. $t_{a}$ is a specific threshold devised to discriminate abnormal *n*-dimensional vectors, and a score of $t_{a}=0.5$ can be confidently used for separating normal and abnormal vectors [31]. After the Isolation Forest (iF) is learned, it is tested with the *n*-dimensional vectors associated with the normalised scaleogram of the current EEG window ($B_{s}+1$) and a score for each is calculated. Those with a value greater than $t_{a}$ are considered abnormal, thus potentially artefactual, and their timestamp is stored in a list AT (anomalous time locations). The power of signals at these abnormal time locations needs to be attenuated. At this stage, the original scaleogram (non-normalised) of the current EEG window is reconsidered, and its normalised version is discarded. This is necessary because the inverse discrete wavelet transform (iDWT) will be applied to the denoised vectors of the current EEG window to reconvert them to the time domain. Such inversion requires a set of concatenated approximation and detailed DWT coefficients. If normalised DWT coefficients across decomposition levels were used, iDWT would lead to a wrong EEG signal reconstruction.

### 3.4. The Expansion Step and the Denoising Strategy

$E_{s}$ is the expansion step used in the final denoising of the DWT coefficients. According to wavelet theory [35], neighbour DWT coefficients are similar in magnitude, so they should also be denoised to obtain a smooth artefact reduction around each anomalous vector and not a sharp one. $E_{s}$ defines how many neighbouring vectors should also be considered for denoising around each vector identified as anomalous. This expansion results in a new list of timestamps for anomalous vectors, $AT_{exp}$. The *onEEGwaveLAD* framework is grounded on an adaptive thresholding mechanism for manipulating DWT coefficients at the different decomposition levels via its sliding buffer. This mechanism begins by computing the medoid $B_{medoid}$ of all the *n*-dimensional vectors in the moving buffer to identify the most representative neural response in the frequency (scale) domain prior to the current EEG window. Subsequently, a mitigator vector Mtg is introduced, which is the complement of the distance of the specific DWT coefficients of a vector *a* in the list $AT_{exp}$, from the medoid $B_{medoid}$ of the buffer $B_{s}$, divided by the maximum distance vector from it, derived from the vectors in the buffer $B_{s}$. Formally,

(7) $$\vec{mtg_{a}}=1-\frac{d(\vec{a},B_{medoid})}{max(d\left(\vec{v},B_{medoid}\right):\;\forall v\in B_{s})}\;\forall a\in AT_{exp}$$

with $\vec{mtg_{a}}:\left[0,1\right]\in ℜ$ the mitigating vector used to denoise every vector *a* in $AT_{exp}$. In simple terms, such a mitigator is softer for vectors close to the medoid (typical neural behaviour) and more aggressive for those far from it (potential artefacts). The new vector $\vec{a_{den}}$ of denoised DWT coefficients of each of the *n*-dimensional vectors in the expanded list $AT_{exp}$ is the element-wise multiplication (Hadamard product) with the mitigator vector. Formally,

(8) $$\vec{a_{den}}=\vec{a}⊙\vec{mtg_{a}}$$

This operation can also cause some of a vector’s DWT coefficients to be reduced more aggressively at one scale (frequency) than others. Future work might include assigning weights to the specific scales (frequencies) according to the type of artefact (blinks or muscle activity).

### 3.5. EEG Single-Channel Recomposition

*onEEGwaveLAD* terminates with a reshaping procedure that concatenates all the non-normalised *n*-dimensional vectors (denoised or not) of the current EEG window, preserving their time appearance (denoised scaleogram of length *n*). Some of these coefficients are repeated $2^{m-1}$ times for each decomposition level *m* and must be removed. At the first decomposition level, with a window of length $RTW_{L}$, and a sampling rate $S_{r}$, $k=(\left(RTW_{L}*S_{r}\right)/1000*0.5)$ DWT coefficients exist; thus, for each decomposition level *m*, only $k/2^{m-1}$ coefficients are unique (not repeated), while the others must be removed. This can be achieved by taking the first DWT coefficient for every $2^{m-1}$ coefficients at a level *m*. This reshaping procedure results in fewer DWT coefficients at higher levels (lower frequencies) and more at lower levels (higher frequencies), with the same shape as the output of Equation (1). These are input into the inverse discrete wavelet transformation (iDWT) (inversion of Equation (1)) for their conversion back into the time domain. Formally,

(9) $$x^{den}\left(t\right)=\sum_{n}C_{M,n}^{den}\Phi M,n\left(t\right)+\sum_{m=1}^{M}\sum_{n}d_{m,n}^{den}\Psi m,n\left(t\right)$$

where $C_{M,n}^{den}=<x,\Phi M,n>$ and $d_{m,n}^{den}=<x,\Psi m,n>$ are, respectively, the sets of denoised approximation and detail coefficients. This eventually returns a signal $x^{den}\left(t\right)$ in the time domain, which is the denoised version of the original.

## 4. Design and Methods

An experiment was used to test the capability of various *onEEGwaveLAD*’s instantiations to denoise muscle artefacts. The particular instantiations are synthesised in Table 1, followed by the research hypothesis, a description of the process of forming the ground truth, and the selected EEG dataset.

### 4.1. onEEGWaveLAD Parameter Setting

Building on the empirical findings of the first instantiation of the framework for ocular artefacts, as given in [3], the following reasoning was applied to set its parameters for the current study. The real-time EEG window length is set to 1 s, with a sampling rate of 1024 Hz. The rationale is to have small EEG windows that can be processed in pseudo-real time and a good amount of time points per window, multiples of a power of 2 (as suggested in Section 3), for a richer analysis. Two mother wavelets are selected: ‘db4’ and ‘Sym4’. The former is from the Daubechies family, with four moments of escape, while the latter is from the Symlet family, with four vanishing moments. Researchers often resort to a trial-and-error approach to choose the mother wavelet, according to the reconstruction error or other metrics, to optimally represent the signal characteristics of the brain’s electrical activity [22,36]. However, this approach is rather time-consuming and was avoided in this research [37]. Also, such mother wavelets were selected because they have been demonstrated to be reasonable for frequency bands decomposition [38].

The buffer capacity was chosen to be 20 windows, which was assumed to be large enough to contain mostly genuine neural activity. Also, such a size was empirically established in a previous instantiation of *onEEGWaveLAD* [3]. The Isolation Forest algorithm’s sub-sampling size was 512, equal to the number of DWT coefficients in a window given the chosen sampling rate. Considering the selected buffer capacity, 10,240 was the number of DWT coefficients in the sliding buffer (512 × 20). The number of IF trees was 100, a reasonable compromise between computational execution time and capacity in learning a robust forest for discriminating anomalies. The anomaly threshold was set to 0.55, which is slightly more stringent than the rule of thumb suggested in the literature [31]. The expansion step was tested against two options to investigate its impact on the denoising capacity: 0 and 5. Muscle artefacts are faster (shorter) than other types of artefacts, such as ocular artefacts. If the boundaries of the current EEG window were exceeded when using an expansion step of 5, then such expansion was clipped to 0 on the left and 512 on the right side.

### 4.2. Research Hypothesis

**Figure 1:**
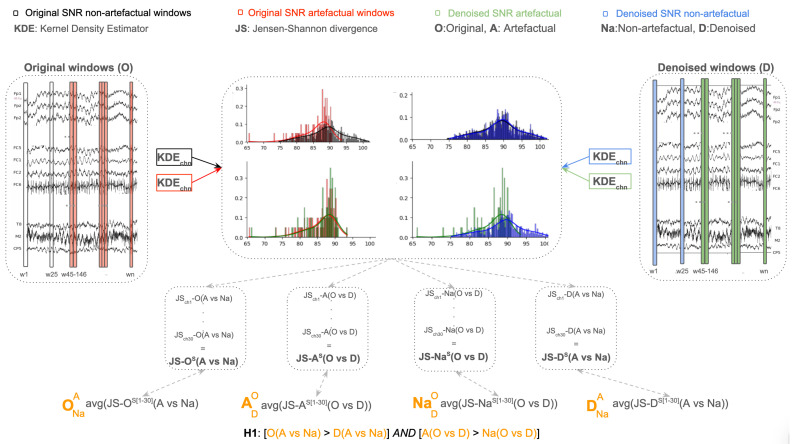
A diagrammatic representation of the research hypothesis.

The research hypothesis tested (Figure 1) was as follows:

**H1.**  *IF an onEEGwaveLAD instantiation is executed in real-time for each subject (37 in ERP-CORE N170 dataset), THEN the average of the Jensen–Shannon distances between the distribution of the signal-to-noise ratios (SNRs) in the artefactual (A) and non-artefactual (Na) windows of the original (O) EEG channels (30) will be higher than those in the denoised (D) EEG channels across subjects $\left[O_{Na}^{A}>D_{Na}^{A}\right]$ AND the average of the Jensen–Shannon distances between the distribution of the signal-to-noise ratios (SNRs) in the original (O) and denoised (D) EEG channels (30) in the artefactual windows (A) will be higher than that calculated in the non-artefactual (Na) windows across subjects $\left[A_{D}^{O}\right)>Na_{D}^{O})]$.*

Informally, the first expectation is that the signal-to-noise ratio of all the EEG channels in the artefactual intervals (windows), identified by the strategy presented in Algorithm A1 (Appendix A), should be naturally lower than in non-artefactual windows. In other words, muscle movements should negatively impact the SNR in the artefactual windows. However, on the other hand, after running an *onEEGwaveLAD* instantiation on all EEG data, the SNR of all the channels in the artefactual intervals should increase because the muscle artefacts should have been mitigated. Technically, the distribution of the 30 SNRs (one for each channel for a subject) in the original artefactual windows should have a higher Jensen–Shannon (JS) divergence than that of the channels in the original non-artefactual windows when compared to the JS divergence between the denoised artefactual versus the denoised non-artefactual windows. This should be observed across all subjects ($\left[O_{Na}^{A}>D_{Na}^{A}\right]$).

The second expectation in the hypothesis is that the improvement in SNR should be significantly higher in the artefactual windows than in the non-artefactual windows across all subjects ($\left[A_{D}^{O}\right)>Na_{D}^{O})]$). This is to test the capability of *onEEGwaveLAD* to denoise only the artefactual windows, and not erroneously the non-artefactual ones, whose signals should not be mitigated. The Jensen–Shannon (JS) divergence was chosen for its ability to accurately measure the similarity between two probability distributions. The goal is to compare the occurrence of random artefacts before and after denoising. Thus, such distributions contain the signal-to-noise ratios of the artefactual and non-artefactual windows across EEG channels. Probabilities were chosen because the number of artefactual windows containing muscle artefacts is much lower than that containing genuine neural signals. Therefore, a probability distribution can better represent and describe the variability and frequency of these two types of signals. The Jensen–Shannon (JS) divergence was preferred over the Kullback–Leibler divergence because, contrary to the latter measure, the former is symmetric and has a finite range. The lower the JS divergence, the more similar the two distributions, while values towards 1 indicate greater dissimilarity [39,40]:

$$JS\left(P||Q\right)=\frac{1}{2}KL(P||\frac{\left(P+Q\right)}{2})+\frac{1}{2}KL(Q||\frac{\left(P+Q\right)}{2})$$

with KL(P||Q) the KL divergence between *P* and *Q*, JS(P||Q), P(x) the distribution of P(x) over x, and Q(x) the distribution of Q(x) over x.

### 4.3. Dataset

To test the research hypothesis, the open-access N170 dataset of the ERP-CORE (Compendium of Open Resources and Experiments) was selected, containing EEG data from 40 participants (25 female, 15 male; mean years of age = 21.5, SD = 2.87, range 18–30; 38 right-handed; native English competence; normal colour perception; normal or corrected-to-normal vision; no neurological injury or disease history) (https://doi.org/10.18115/D5JW4R, accessed on 15 May 2025). Participants were exposed to a visual discrimination paradigm for isolating the face-specific N170 response, and three were discarded due to excessive artefact contamination by visual inspection [41]. EEG data, with an average length of 581 s, with 55 standard deviations, was recorded using a Biosemi ActiveTwo recording system with active electrodes (FP1, F3, F7, FC3, C3, C5, P3, P7, P9, PO7, PO3, O1, Oz, Pz, CPz, FP2, Fz, F4, F8, FC4, FCz, Cz, C4, C6, P4, P8, P10, PO8, PO4, O2) placed following the 10/20 standard. P01 was assigned to the common-mode-sense (CMS) electrode, with the driven right leg (DRL) electrode at PO2. Signals were low-pass-filtered using a fifth-order sinc filter with a half-power cutoff at 204.8 Hz and then digitised at 1024 Hz with 24 bits of resolution. Further details on data collection are in [42].

### 4.4. Ground Truth Formation

A ground truth was formed to assess the capability of *onEEGwaveLAD* in detecting and removing muscle artefacts with its specific instantiation (Table 1). Such ground truth is in the form of a set of intervals likely to contain muscle artefacts, and it is generated by the offline Algorithm A1 (Appendix A). Firstly, a band pass filter within the range of frequencies [lFreq,hFreq] is executed on the multi-channel EEG data multiChData. The filtered data multiChFiltData is then transformed via the Hilbert transformation, and the absolute value of its analytical signal, the envelope, is computed (multiChEnvelope). The envelope’s Z-scores for each of the *n* channels are calculated, then summed across channels and divided by sqrt(n). These integrated scores (integratedArtScores) are low-pass-filtered at 4 Hz to prevent false-positive transient peaks. The filtered integrated scores (filArtScores) that exceed the specified z-score threshold ($ZS_{th}$) are preserved, and their index is saved (along with the time location). The parameter minLen is a cutoff value for whether short intervals of good neural data between muscle artefacts are included in the surrounding artefactual intervals. The identified artefactual intervals starting from each saved index (artMaskLocs) are computed (artIntervals). These are portions of EEG data likely containing muscle artefactual activity, and their starting index and duration are returned. According to [4], muscular artefacts generally occur above 30 Hz and up to 300 Hz. However, they are more prominent in the range [110, 140] Hz, a practical heuristic often used by neuroscientists (mne.tools/stable/auto_examples/preprocessing/muscle_detection.html, accessed on 15 May 2025). For this reason, the parameters lFreq and hFreq were set to 110 Hz and 140 Hz, respectively. The parameter minLen represents the shortest allowed duration of ‘good data’ (in seconds) between adjacent annotations, and it was set to 0.1. Ultimately, selecting the threshold $ZS_{th}$ is not straightforward, as noted in the literature [4]. Tuning this threshold can result in more muscle artefactual intervals being detected, which can lead to false positives, or fewer intervals, which can negatively impact true positives. Consequently, a procedure was designed to invoke Algorithm A1 (Appendix A) with different increasing ZS-th thresholds to identify real true positives. This procedure includes a strategy to filter false positives. Such a strategy is grounded on the evidence that muscle artefacts are identifiable based on duration, morphology, and firing rate. In detail, the high-frequency power spectrum is higher in intervals with muscle artefacts than in those without them [43], and the potentials generated in the muscles are of shorter duration than those generated in the brain [4].

The denoising strategy begins by computing the spectrum of the overall multi-channel EEG data for a given participant using the Welch method within the frequency range [30, 300] Hz. The resulting spectral densities are averaged across channels, and the power of this average is computed. A set of thresholds for Z-scores is iterated, between 5 and 2, with a step of −0.1. At each iteration, Algorithm A1 (Appendix A), which aims to find muscle artefacts, is invoked on the subject’s multi-channel EEG data, with a frequency range [110, 140], a current threshold, a minimum length of 0.1 s between artefactual intervals, and 30 channels. For each identified artefactual interval in the current iteration, the power spectrum is computed using the Welch method for frequencies within the range [30, 300] Hz. The average of the returned spectral densities is computed across channels, and its power is calculated. Subsequently, the one-tailed Mann–Whitney parametric statistical U-test is used to compare the two distributions containing the power spectral densities: those computed for the entire EEG data for a subject and one calculated for the current artefactual intervals, with an alpha value of 0.01. In detail, the alternate hypothesis is that the former distribution (all EEG data) is stochastically less than the latter distribution (artefactual interval data). If the resulting *p*-value of such a test is less than the selected alpha value, the null hypothesis will be rejected in favour of the alternative hypothesis. This demonstrates how the artefactual interval’s power spectral densities are statistically significantly higher than those representing the entire EEG data. If this is the case, such an interval is deemed legitimate and appended to a container of valid intervals. At each iteration, a ratio of valid intervals over all the possible intervals for the current tested threshold is computed. It is saved if this is higher than the previous iteration, and the corresponding threshold is memorised as ideal. When the loop stops, Algorithm A1 is invoked again with such an ideal threshold because it maximises precision, the true positives, and legitimate intervals affected by muscle-generated myogenic potentials.

## 5. Findings and Discussion

**Figure 2:**
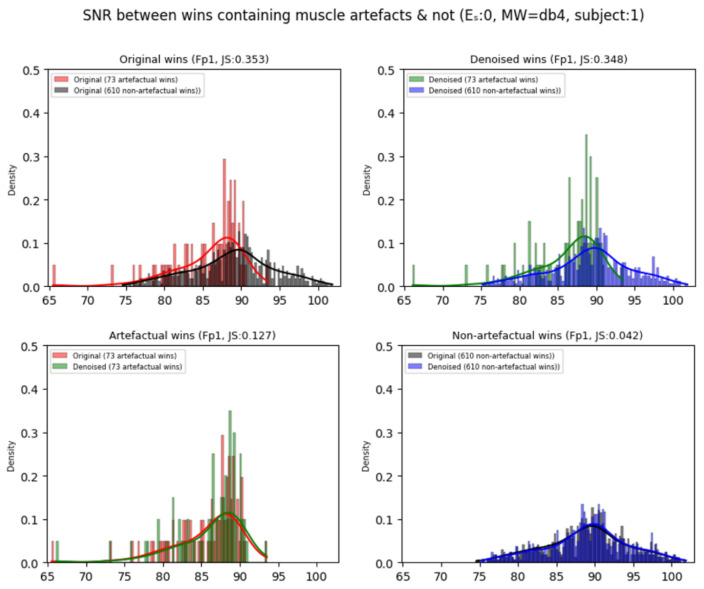
Comparison of the probability distributions of the signal-to-noise ratios in the original and denoised artefactual and non-artefactual windows.

**Figure A1:**
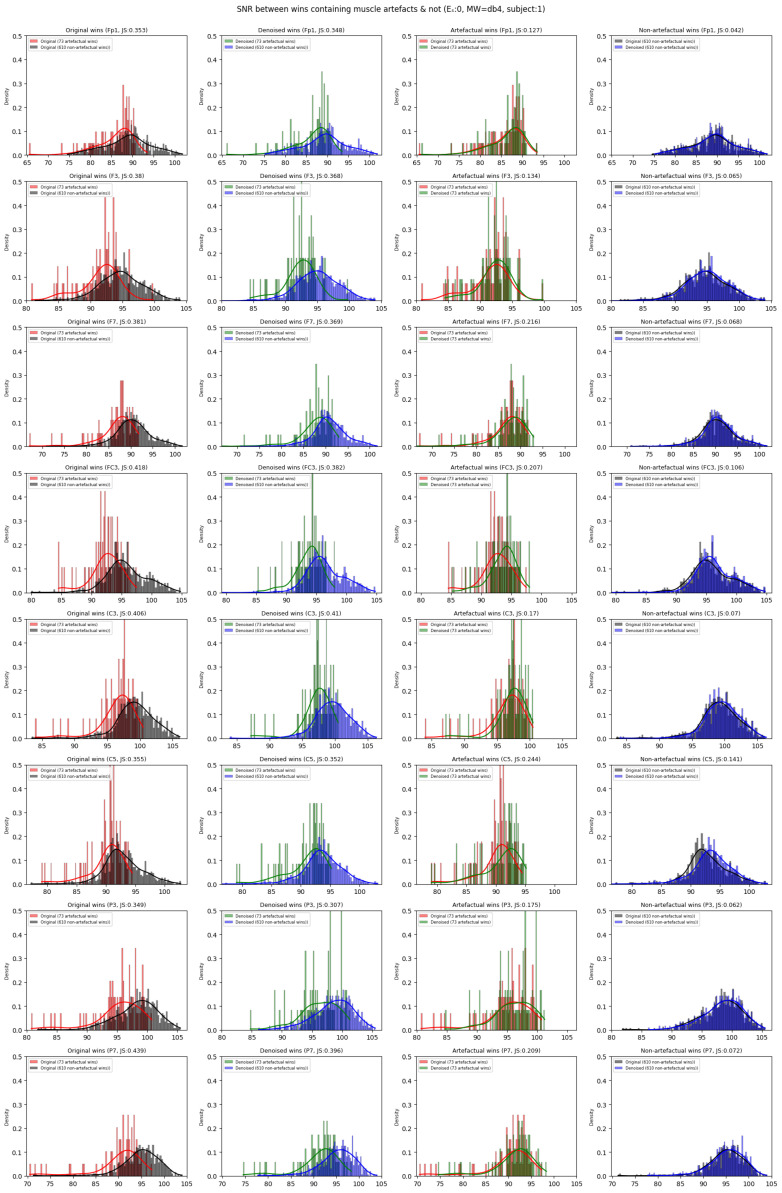

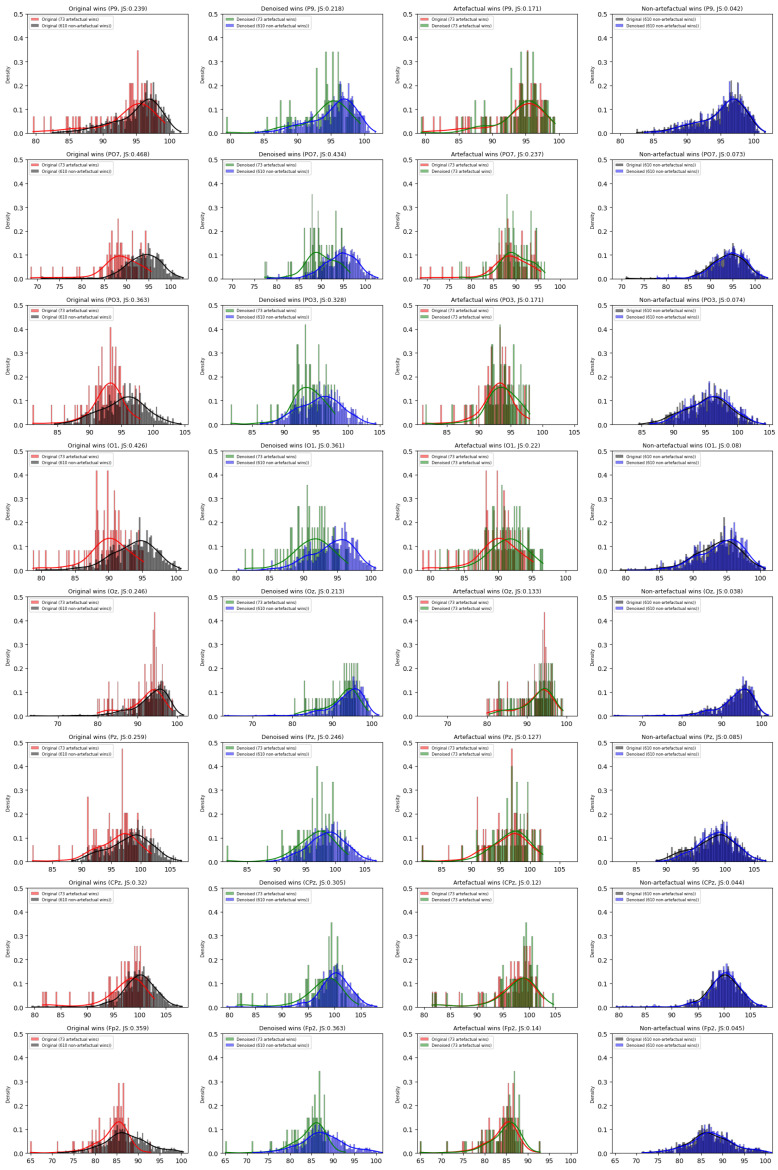
Comparison of the distributions of the signal-to-noise ratios for a subject in the artefactual and non-artefactual windows, on the original and denoised signals, grouped by EEG channels (Fp1, F3, F7, FC3, C3, C5, P3, P7, P9, PO7, PO3, O1, Oz, Pz, Cpz, Fp2).

**Figure A2:**
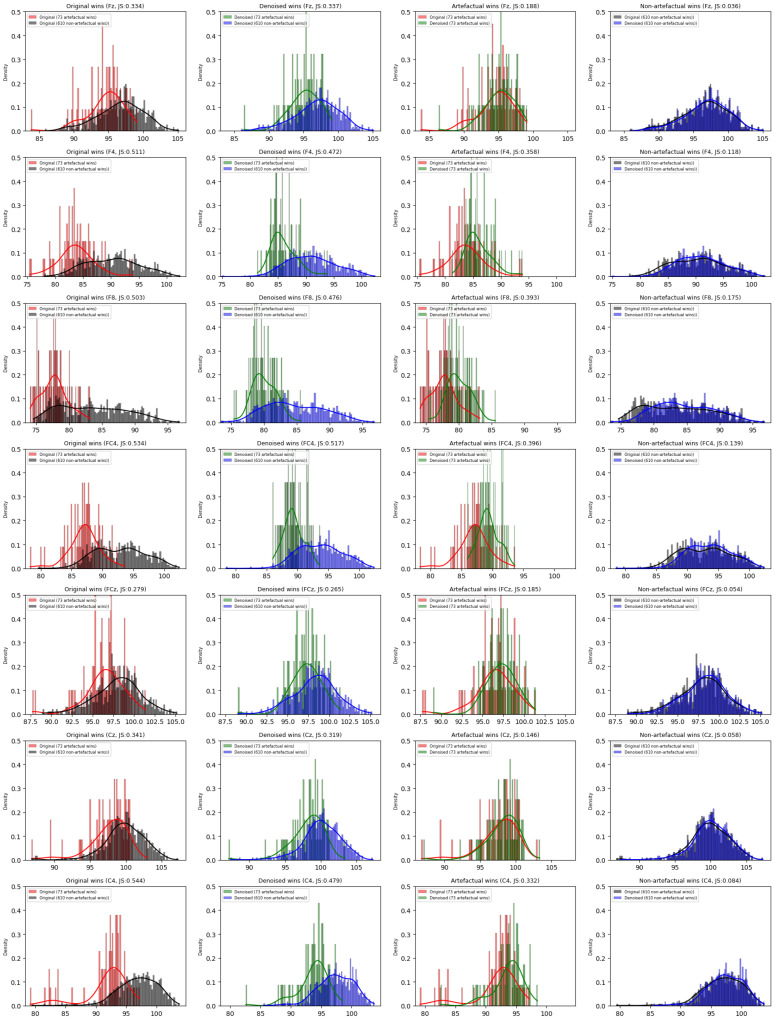

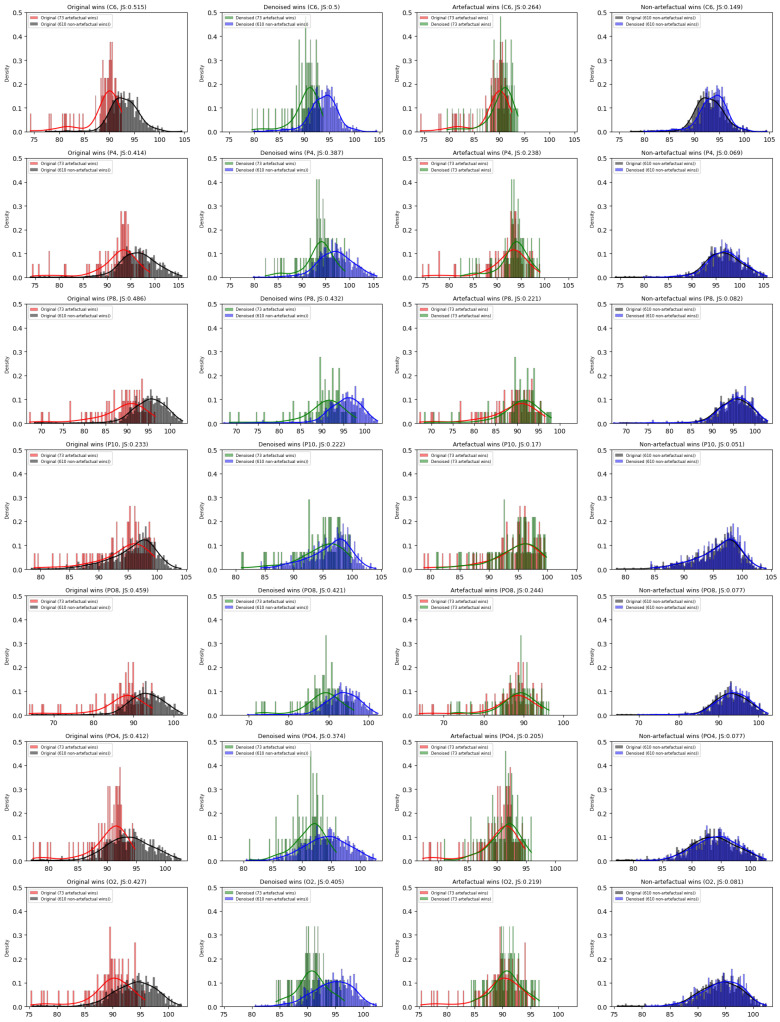
Comparison of the distributions of the signal-to-noise ratios for a subject in the artefactual and non-artefactual windows, on the original and denoised signals, grouped by EEG channels (Fz, F4, F8, FC4, FCz, Cz, C4, C6, P4, P8, P10, PO8, PO4, O2).

Figure 2 depicts the comparison of the probability distributions of the SNRs of the original Fp1 signal between the artefactual and non-artefactual windows (first column), and in the denoised signal (second column), for subject 1. It also compares the original and denoised signals in the artefactual (third column) and non-artefactual windows (fourth column). Figure A1 and Figure A2 (Appendix B) depict the results for all the other channels. Generally, the trends that emerge in these pictures are shared across all 30 participants (too many to display). As observed, each original signal’s probability distribution is always shifted between the artefactual and non-artefactual window (column 1, Figure 2). This confirms that the SNR in the non-artefactual windows (black) is higher than in those containing muscle movements (red) (as identified by Algorithms A1 and A2 in the Appendix A). Each subject has a different number of artefactual and non-artefactual windows (for example, for subject 1, 73 are artefactual, 610 are non-artefactual).

**Figure 3:**
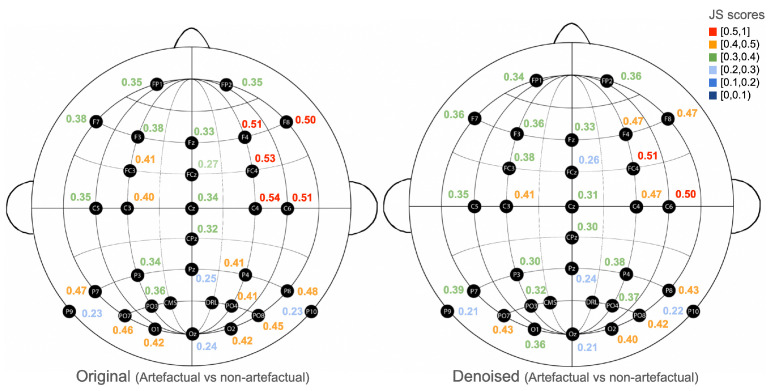
Comparison of the Jensen–Shannon divergences of the signal-to-noise ratios between the artefactual and non-artefactual windows, grouped by channel (**left**: original; **right**: denoised) for subject 1.

Concerning the denoised signal’s probability distributions (column 2, Figure 2), the SNRs of the artefactual windows (green), after applying the *onEEGwaveLAD* framework (with expansion step: 0, and MW: db4), have been shifted more towards the SNRs of the non-artefactual windows (blue), as confirmed by the reduced Jensen–Shannon (JS) divergence (0.348<0.353). As depicted in Figure 3, most JS scores across channels (left) are lower in the denoised version (right). This suggests that signal denoising in the artefactual windows had a positive effect, as their SNR increased, thereby reducing the distance (divergence) to their non-artefactual counterparts.

**Figure 4:**
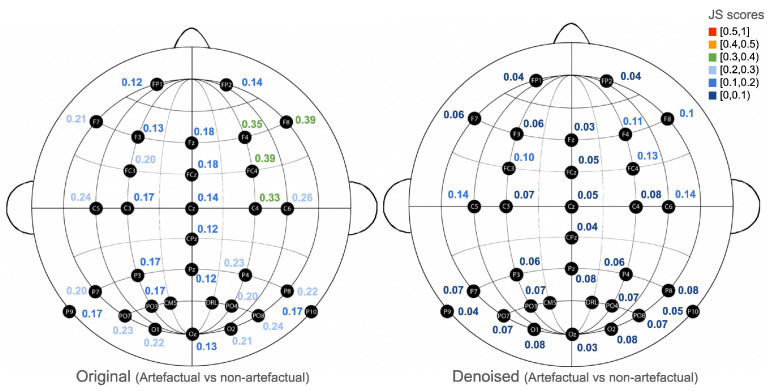
Comparison of the Jensen–Shannon divergences of the signal-to-noise ratios between the original and denoised windows, grouped by channel (**left**: artefactual; **right**: non-artefactual) for subject 1.

Someone can argue that this is not true because the SNRs of the signals in the counterfactual windows might have remained the same, while they might have decreased in the non-artefactual windows, thus reducing their distance. However, as Figure 4 demonstrates, this is not the case. In fact, the JS divergences of the SNRs of artefactual versus non-artefactual windows in the original EEG channels (left) are always higher than those in the denoised EEG channels (right). This demonstrates that the *onEEGwaveLAD* instantiation effectively mitigates artefactual windows, thereby increasing their signal-to-noise ratio while leaving non-artefactual windows intact, with minimal modifications. For subject 1, this reduction of noise is prominent in the right part of the frontal cortex (F8, F4), the right central (C4, C6), and the area between them (FC4).

**Figure 5:**
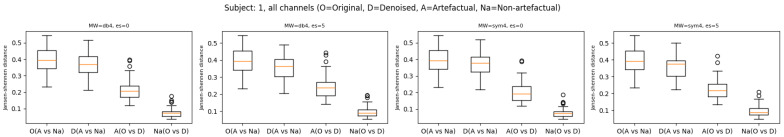
Box plots of the Jensen–Shannon divergences of the signal-to-noise ratios of the artefactual (A) and non-artefactual (Na) windows on the original (O) and denoised (D) EEG channels, across four instantiations of the *onEEGwaveLAD* framework for subject 1.

**Figure A3:**
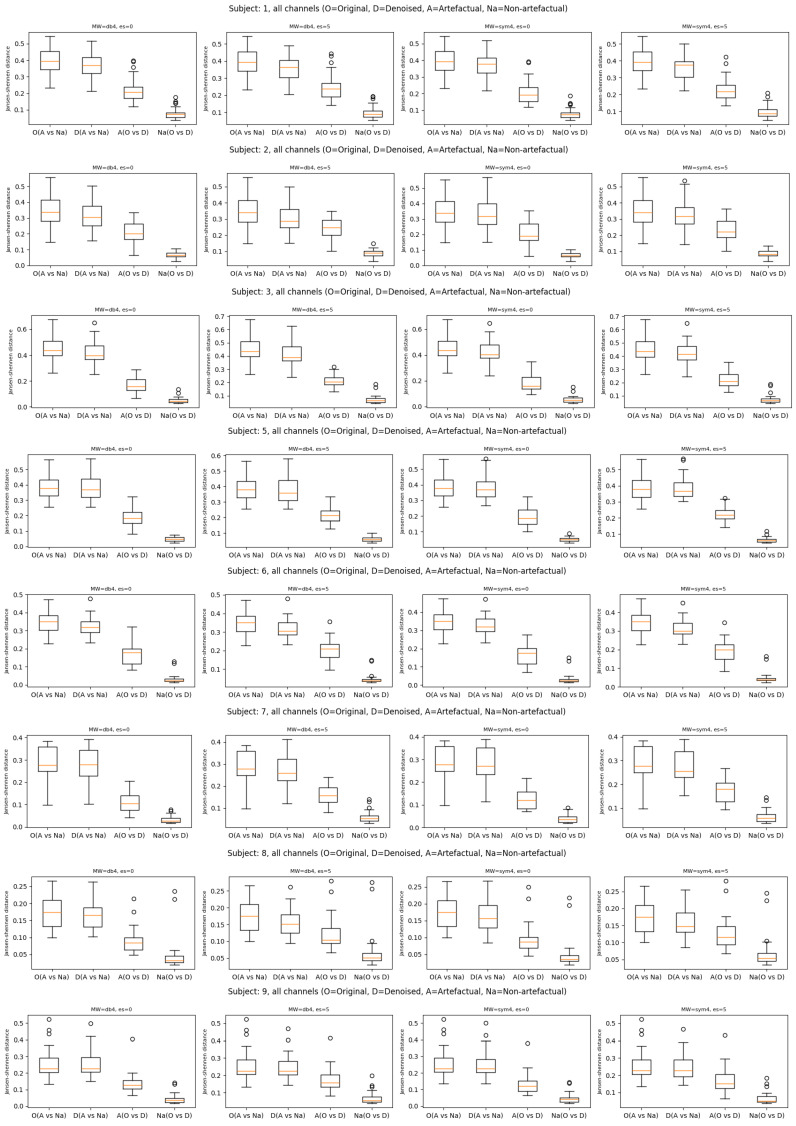

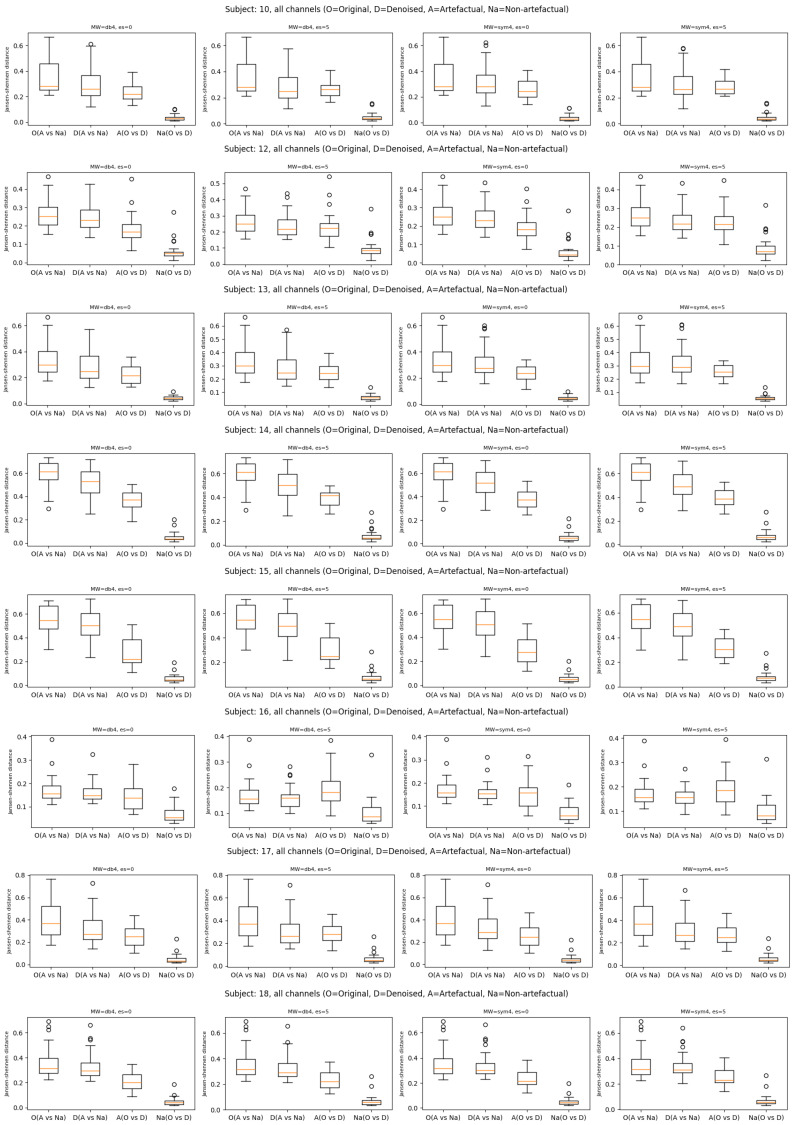
Box plots of the Jensen–Shannon divergences of the signal-to-noise ratios of the artefactual (A) and non-artefactual (Na) windows on the original (O) and denoised (D) EEG channels, across four instantiations of the *onEEGwaveLAD* framework (subjects: 1–10, 12–18).

**Figure A4:**
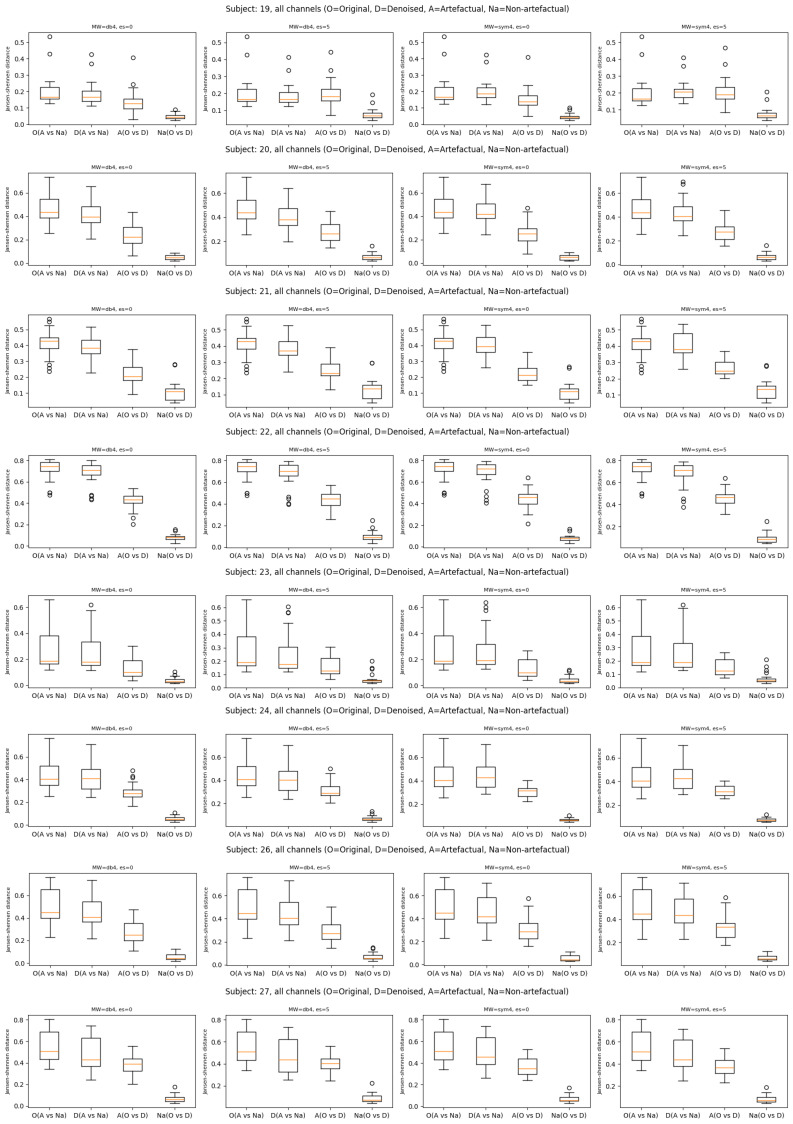

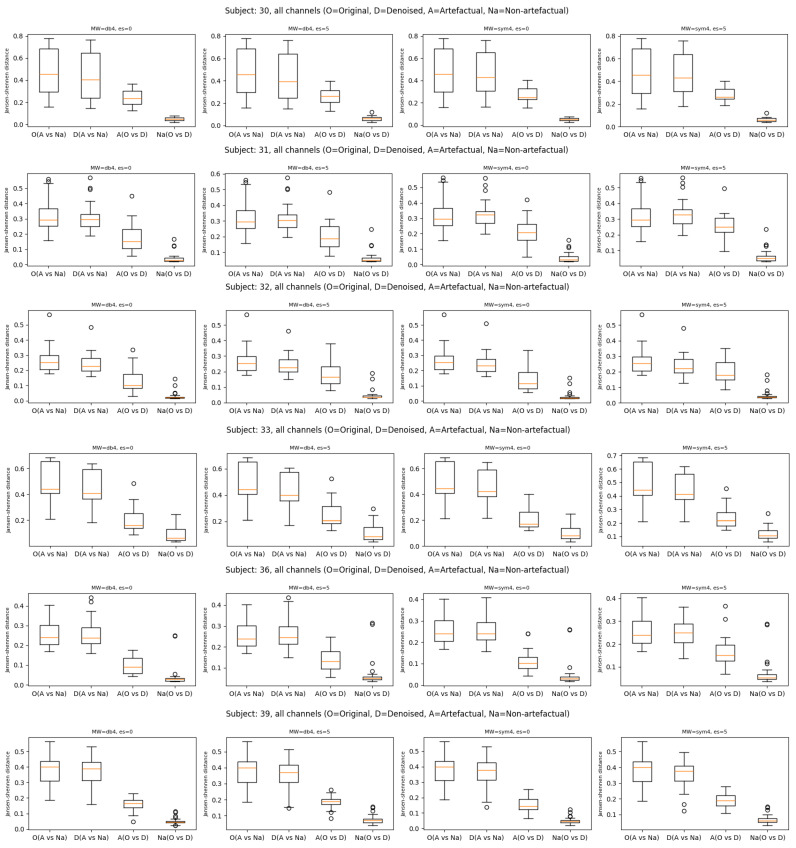
Box plots of the Jensen-Shannon divergences of the signal-to-noise ratios of the artefactual (A) and non-artefactual (Na) windows on the original (O) and denoised (D) EEG channels, across four instantiations of the *onEEGwaveLAD* framework (subjects: 19–24, 26, 27, 30–33, 36, 39).

These results are also confirmed by the box plots in Figure 5 of the four different instantiations of the *onEEGwaveLAD*. Reasonably, the first part of the research hypothesis is that $\left[O_{Na}^{A}>D_{Na}^{A}\right]$ can be accepted. Regardless of the specific instantiation, the second box plot D(AvsNA) (denoised JS divergences) is always lower than the box plot O(AvsNa) (original JS divergences). This lack of significant difference was expected because the results of all the channels in all the windows were considered together. However, for the second part of the hypothesis, $\left[A_{D}^{O}\right)>Na_{D}^{O})]$, the results are clearly different. The last box plot (extreme right, Figure 5) of each instantiation of *onEEGwaveLAD* (each of the four charts) has very low JS scores compared to those associated with the artefactual windows (third from left). This demonstrates how the non-artefactual windows have been correctly identified and minimally denoised, given the lower standard deviation. Note that minimal denoising, with a resulting increase in SNR in the non-artefactual windows, is somehow expected because other types of artefacts, such as ocular or cardiac, could be present and might have been denoised. Noticeably, in Figure A3 to Figure A4 (Appendix C), the tendencies that emerged for subject 1 are highly similar across all the subjects.

**Figure 6:**
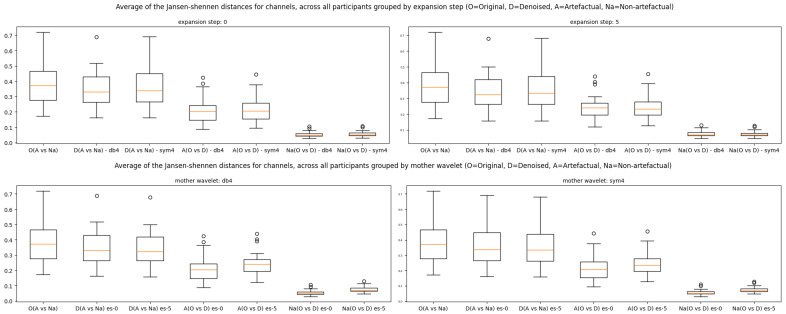
Box plots of the Jensen–Shannon divergences of the signal-to-noise ratios of the artefactual (A) and non-artefactual (Na) windows of the original (O) and denoised (D) EEG channels, across four *onEEGwaveLAD*’s instantiations for all subjects grouped by expansion step ($E_{S}$), and mother wavelet (MW).

Figure 6 depicts all subjects’ overall results, grouped by expansion step and mother wavelet. Using the “db4” and “sym4” wavelets did not lead to significant differences in the JS scores (second vs. third box plot, fourth vs. fifth, and sixth vs. seventh of the top quadrants), with expansion steps 0 and 5. This suggests that both had the same capacity to identify transient variations within the EEG signals and were potentially interchangeable. Expansion steps 0 and 5 also did not significantly affect artefact mitigation (second vs. third box plot, fourth vs. fifth, and sixth vs. seventh of the bottom quadrants) across the mother wavelets. This suggests that expanding the neighbourhood around each identified anomalous time location does not improve artefact mitigation. This is likely because muscle movements are very rapid, with high frequencies; thus, mitigating the signals around them is unnecessary. However, all the quadrants of Figure 6 confirm the *onEEGwaveLAD* instantiations’ capability to identify the artefactual (fourth/fifth box plots) and non-artefactual windows (sixth/seventh) correctly, given that the signals’ mitigation is significantly lower in the latter cases.

### In-Depth Within-Window Analysis on Denoising Capacity

**Figure 7:**
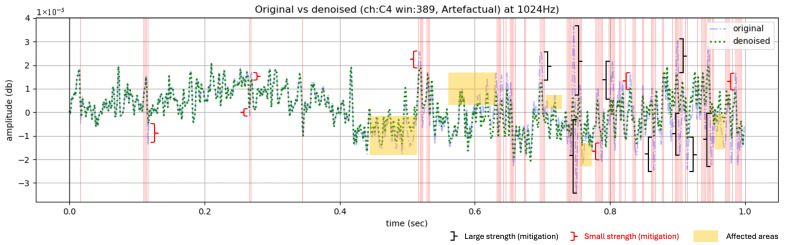
An EEG window with artefactual locations (red vertical lines) with large/small mitigation and the effect of denoising on neighbours (yellow areas).

**Figure A5:**
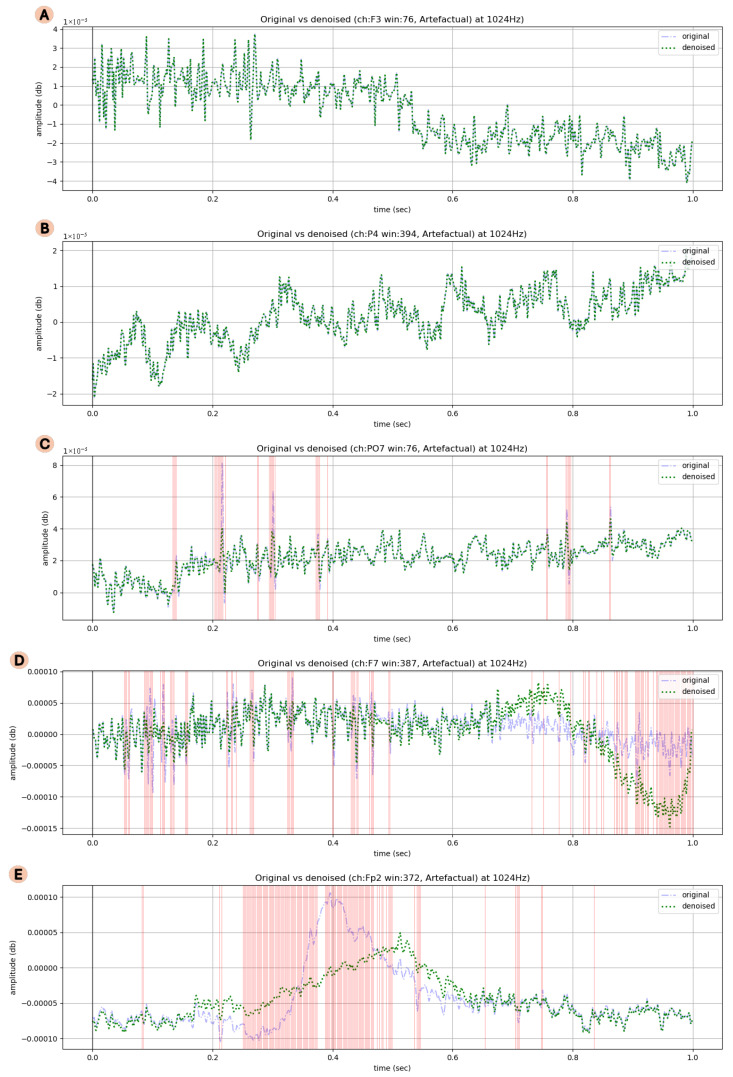

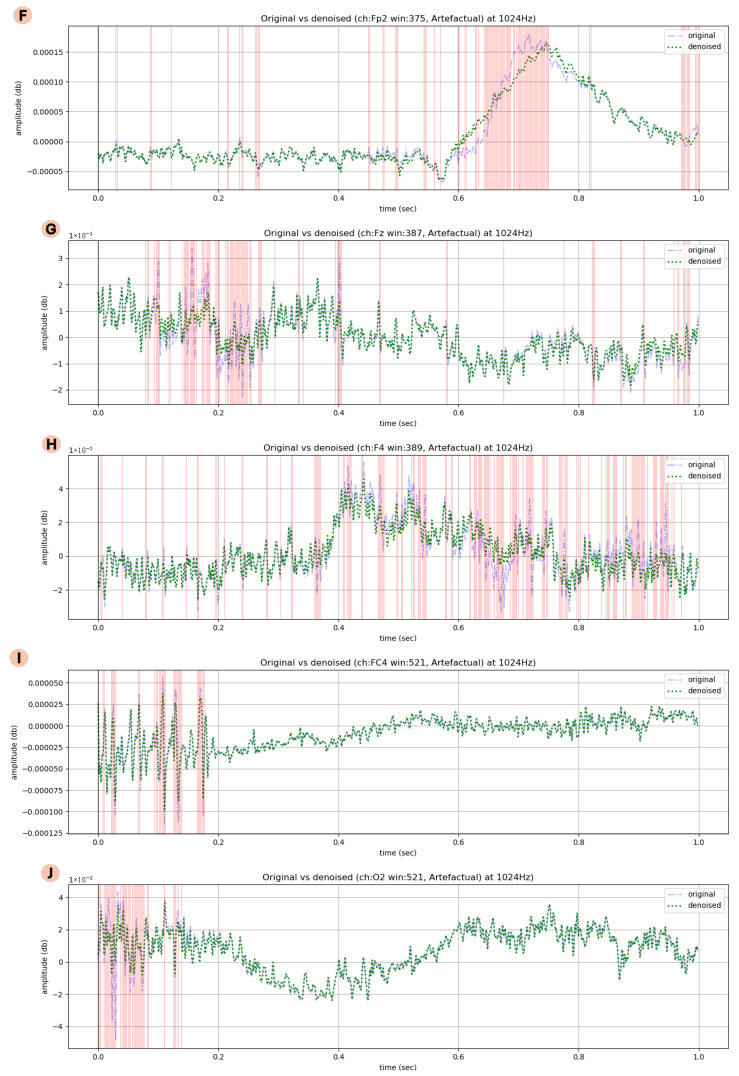
Examples of random artefactual windows for subject and random channels, with original and denoised signals, with vertical lines at the time locations deemed anomalous by the *onEEGwaveLAD* instantiation. (**A**,**B**): correctly preserved windows by *onEEGWaveLAD*, despite being deemed artefactual by the offline ground truth algorithm; (**C**): transient spikes successfully identified and removed by *onEEGWaveLAD*; (**D**): correctly identified spikes by *onEEGWaveLAD* but with hampered reconstruction due to the issue of the cone of influence; (**E**): a correctly identified blink by *onEEGWaveLAD*, and moderate mitigation; (**F**): a correctly identified blink by *onEEGWaveLAD* with minimal mitigation; (**G**,**H**): scattered transient spikes correctly identified and mitigated by *onEEGWaveLAD*; (**I**,**J**): muscular activity successfully identified and mitigated by *onEEGWaveLAD* at the start of the window.

**Figure A6:**
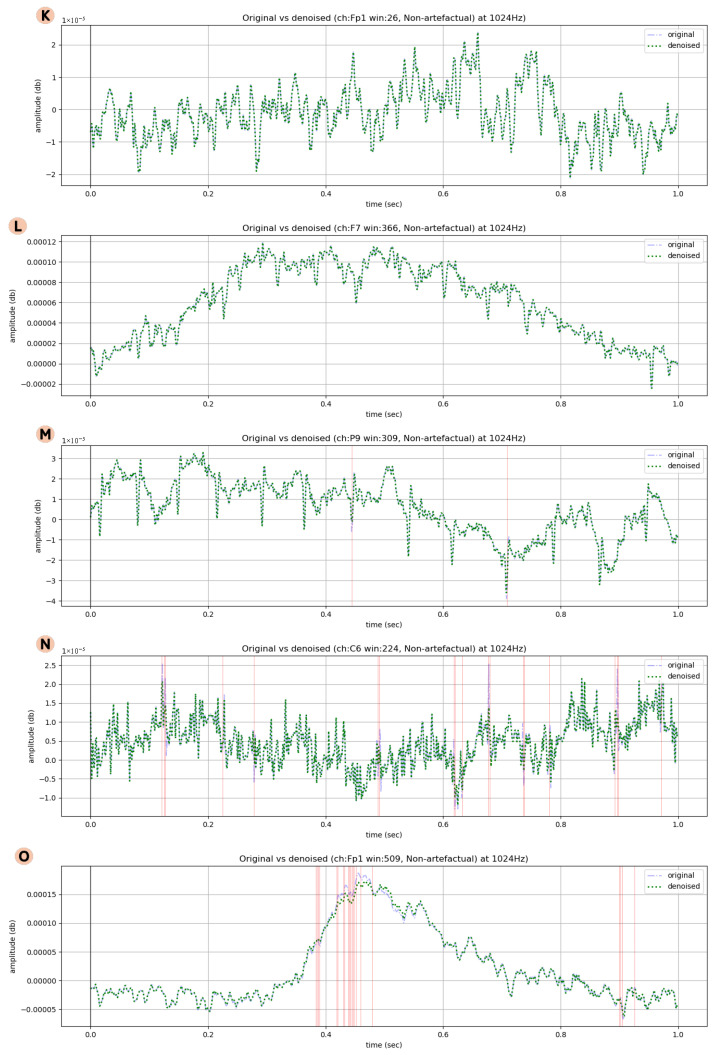

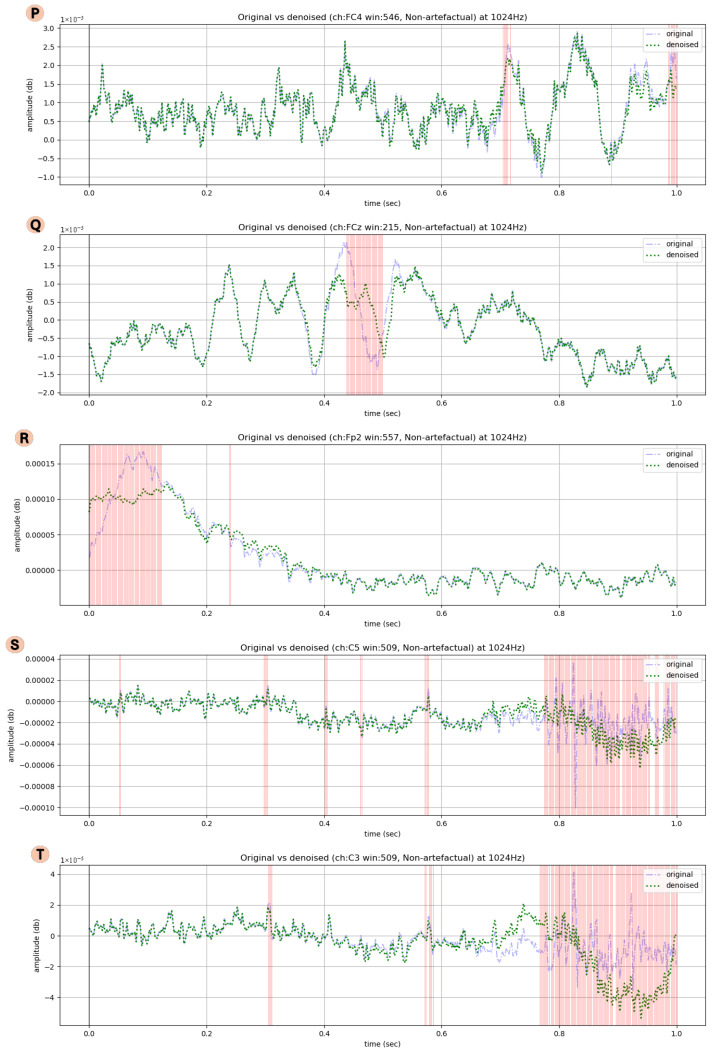
Examples of random non-artefactual windows for subject and random channels, with original and denoised signals, with vertical lines at the time locations deemed anomalous by the *onEEGwaveLAD* instantiation. (**K**,**L**): correctly non-artefactual windows identified by *onEEGWaveLAD* and successfully preserved; (**M**,**N**): rare transient peaks identified by *onEEGwaveLAD* but not the offline ground truth algorithm; (**O**): a missed blink not identified by *onEEGwaveLAD* and the offline ground truth algorithm; (**P**,**Q**): rare transient activity identified by *onEEGwaveLAD* and marginally mitigated; (**R**): blink correctly identified by the *onEEGwaveLAD* and correctly mitigated, despite not deemed artefactual by the offline ground truth algorithm; (**S**,**T**): correctly identified artefacts by *onEEGwaveLAD* at the end of the window, but hampered reconstruction due to the issue of the cone of influence.

To gain a deeper understanding of how the artefacts were mitigated by the adaptive thresholding mechanism for DWT coefficients mitigation at the decomposition levels of *onEEGwaveLAD* (Section 3, Equations (7) and (8)) with the specific instantiations of Table 1, some random artefactual and non-arterfactual windows (Figure A5 and Figure A6) for participants were picked (too many to depict: on average 600 EEG windows for each of the 30 subjects with 30 channels each). After a thorough visual examination, the rationale was to select random EEG windows with heterogeneous waveforms, varying degrees of noise contamination, and channels that best represent the full range of variations of the original single channels and their denoised versions. Each of these windows is similar to that in Figure 7, displaying the original signal overlapped by its denoised version for a one-second EEG segment for a single channel with some artefacts (red vertical lines) identified. At these times, the effects of the denoising strategy can be observed by comparing the original signal with the denoised signal: the tall transient spikes have been reduced in magnitude towards zero, with different strengths. Note that such strengths (small or large) depend on the denoising strategy defined in Equations (7) and (8), which operate not directly on time series but in the DWT-coefficient domain. The farther their multi-dimensional vectorial representation in such a domain is from the medoid (vector in the middle of the moving buffer), the stronger the attenuation towards it. Additionally, since the inverse discrete wavelet transformation uses a mother wavelet that is shrunk and stretched at each decomposition level over time, this also affects the signal’s reconstruction around the anomalous time locations, with reduced DWT coefficients (yellow areas).

The first two top charts, A and B (Figure A5, Appendix D), are examples of windows that have not been denoised despite being categorised as artefactual. This can happen because the offline multi-channel algorithm, used for identifying contaminated areas of the overall EEG data (Algorithm A2, Appendix A), may have based its decision on the degree of muscle artefact contamination in other channels within the same window. Therefore, this total absence of denoising is intuitively explainable because *onEEGwaveLAD* operates on a single channel, unaffected by the information from other channels. In chart C, some artefactual activities, with transient peaks of higher magnitude, have been successfully found and mitigated. In chart D, more artefactual activity has been correctly identified, especially at the end of the window. Specifically, transient peaks have been successfully mitigated in the first half of the window. Still, the part towards its end presents a problem in denoising since the underlying signal was severely negatively changed, amplifying the noise. This is likely explained by edge effects and the cone-of-influence phenomenon described in Section 3. In such a specific circumstance, the application of mother wavelets near the edges of an EEG window inevitably extends their domain outside it, hampering the computation of the DWT coefficients. Therefore, denoising such wrongly computed DWT coefficients leads to an erroneous reconstruction of the signal in the time domain. Acknowledging this is an open problem, it is recommended that an appropriate solution be developed in future work. Charts E and F are examples of artefactual windows where ocular muscular activity is likely. The shape of the original Fp2 signal resembles that of a blink. Here, *onEEGwaveLAD* successfully identified such anomalous activity, given the many anomalous vectors adjacent to each other. However, in chart E, on the one hand, such a blink was significantly reduced in magnitude, but, on the other hand, it was barely mitigated in chart F.

Intuitively, the denoising strategy found no significant deviation of each multi-dimensional vectorial representation of the signal in the DWT domain at the anomalous time locations from the medoid in the moving buffer. Likely, this is because a blink is slower in time (100/200 ms to 400/700 ms) than muscle artefacts and does not exhibit transient activity in the frequency domain. In such a case, only mitigating DWT coefficients at those decomposition levels covering blink frequencies ([2–12/15 Hz]) might decrease the EEG signal’s magnitude while preserving the faster EEG dynamics. It is recommended that this hypothesis be tested in future work. Charts G and H are examples of how *onEEGwaveLAD*, with its instantiations, successfully detected many anomalous time locations, as it was supposed to, since these charts are considered artefactual by the offline muscle detection algorithm (Algorithm A2, Appendix A). It also demonstrated its great denoising capacity, with small and large effects only in these locations, and not elsewhere. Finally, charts I and J demonstrate how anomalous activity was successfully identified at the beginning of the window, and the denoising strategy worked perfectly here, exhibiting no edge effects. In other words, concatenating the EEG window before those depicted in charts I and J (as mentioned in Section 3) as input to the discrete wavelet transformation helped generate DWT coefficients that were not affected by edge effects since the cone of influence was larger.

The first top charts K, L (Figure A5, Appendix D) depict windows that have not been identified as containing muscular artefactual activity by both the *onEEGwaveLAD* instantiations and by the offline algorithm (Algorithm A2, Appendix A). Visually inspecting these windows, it seems that no transient activity with high magnitude exists, and therefore, is correctly not identified by *onEEGwaveLAD*. Charts M and N depict some rare transient activity, correctly identified by the *onEEGwaveLAD* instantiation (red vertical lines), with corresponding mitigation. On the one hand, in M, some peaks were identified as anomalous, even if they were small in magnitude. Their modest magnitude reduction is likely due to their inclusion in a vector of DWT coefficients identified as anomalous, but not very distant from the medoid. On the other hand, in N, there was some transient activity with a higher magnitude, which was correctly mitigated with a higher strength and subsequent, more substantial mitigation. This highlights the adaptivity of the mitigation strategy of the *onEEGwaveLAD* framework. In chart O, anomalous activity around 0.4 s was detected, visually mimicking a blink (Fp1 channel). However, this was not mitigated significantly, probably because the selected mother wavelet did not accurately resemble the prototypical waveform of a blink. Therefore, when convolved with the original signal using the discrete wavelet transformation, it did not yield remarkably high DWT coefficients. Consequently, even if those DWT vectors at these time locations were considered anomalous, they were not far from the medoid, resulting in marginal mitigation. Charts P and Q again show some marginal artefactual activity identified, with two degrees of mitigation: the former had low mitigation, and the latter was more pronounced. Chart R depicts a blink identified by the *onEEGwaveLAD* instantiation at the beginning of the window. Here, the signal was correctly mitigated, and the edge effect on the left side of a window did not occur. However, such an effect is evident in charts S and T, where artefactual activity occurred at the window’s end. On the one hand, in chart S, there was a sizeable transient peak of activity around sim0.85 second that was correctly mitigated with high strength. However, in chart T, two large higher-magnitude peaks were spotted (at ∼0.87 and ∼0.9 s). The first was clearly in the cone of influence and correctly mitigated, while the second was not, leading to an erroneous signal reconstruction.

In summary, demonstrating how different instantiations of the *onEEGwaveLAD* framework can be created and tested has shown how muscle artefacts can be confidently identified in an online, fully automated manner. Similarly, the denoising strategy has demonstrated promising capability for various artefacts with different magnitudes. An open existing problem, as already mentioned in Section 3, is associated with artefactual activity found outside the cone of influence, especially on the right side of an EEG window, which needs to be denoised. The DWT transformation, using the strategy mentioned in Section 3, and its inversion (inverse DWT) were ineffective in some cases, because the DWT coefficients had to be estimated outside of this window. However, such improper EEG signal reconstruction occurred in a very limited number of EEG windows and channels, not impacting the overall results. An additional study will be entirely devoted to the design of a more effective strategy and its evaluation.

## 6. Conclusions

The development of online, pseudo-real-time denoisers for EEG signals poses a significant challenge in neuroscience [6,44,45]. This is because multi-channel EEG signals must be processed without supervision in real time, immediately after their recording, requiring significant computational capabilities at various phases. These include transforming the EEG time series into various domains, such as time–frequency, automatic artefact identification in consecutively collected small EEG windows, efficient denoising strategies, and reconstruction of EEG signals in the time domain. This research applied *onEEGwaveLAD*, a fully automated online EEG wavelet-based learning adaptive denoiser for artefact identification and mitigation in EEG signals [3]. It is fully automated because it does not require human intervention in data analysis and processing. It is online because it can process consecutive small windows of EEG data. It is based on the discrete wavelet transform for acquiring richer time–frequency features of EEG signals, with a linear time complexity (O(n)). It is grounded on the Isolation Forest algorithm to identify anomalous time activity in feasible time complexity ($O(n*log_{n}$) from an adaptive sliding buffer of DWT coefficients. It is based on a denoising strategy that penalises anomalous activity according to its distance to the medoid of such a buffer. The findings demonstrate how instances of the *onEEGwaveLAD* can discriminate EEG intervals contaminated by muscle artefacts with a promising capability and leave the other intervals almost intact.

Future work will focus on instantiating novel instances with ad hoc, asymmetric mother wavelets tailored explicitly to other types of artefacts, including ocular and cardiac. It will also include refining the denoising strategy of the DWT coefficients in the moving buffer and investigating the reasonable assumption that such a buffer is artefact-free. Work will focus on comparing the denoising power of *onEEGwaveLAD* with other offline and real-time denoising techniques, including the FASTER [2] and Artefact Subspace Reconstruction techniques [46,47], in terms of signal-to-noise ratio, peak signal-to-noise ratio, power spectral density, and convergence of event-related potential formation. Finally, further tests will be performed with data collected in other contexts, including ecological settings, and not necessarily lab-based settings.

## Figures and Tables

**Table 1:** Parameters of instantiations of the *onEEGwaveLAD* framework for identifying and reducing muscle artefacts.

Description	Parameter	Value
*Real-time EEG window length*	$RTW_{L}$	1000 ms
*Sampling rate*	$S_{r}$	1024 Hz
*Mother wavelet*	MW	[db4, sym4]
*IF sub-sampling size*	$IF_{S}$	512 samples
*Number of IF trees*	$IF_{t}$	100 trees
*Buffer capacity*	$B_{s}$	20 windows
*Anomaly threshold*	$t_{a}$	0.55
*Expansion step*	$E_{s}$	[0, 5]

## Data Availability

No new data was created or analyzed in this study.

## Appendix A. Ground Truth Formation for Muscle Intervals

**Algorithm A1** Muscle artefactual interval detection algorithm.
**procedure** getMuscleArtIntervals(multiChData, lFreq, hFreq, $ZS_{th}$, minLen, *n*) $∥\begin{array}{c} multiChFiltData \end{array}∥\leftarrow bandPassFilter\left(multiChData,lFreq,hFreq\right)$ $∥\begin{array}{c} multiChEnvelope \end{array}∥\leftarrow abs\left(HilbertTransform\left(multiChFiltData\right)\right)$ $∥\begin{array}{c} zArtScores \end{array}∥\leftarrow zScore\left(multiChEnvelope\right)$ [integratedArtScores]←sum(zArtScores)/sqrt(n) [filArtScores]←lowPassFilter(integratedArtScores,4) $\left[artMaskLocs\right]\leftarrow indexesOf\left(filArtScores>ZS_{th}\right)$ [artIntervals]←extractArtSegments(artMaskLocs,minLen) **return** [artIntervals] ▹ Each interval has a starting index and a duration **end procedure**

**Algorithm A2** Plausible muscle artefactual interval detection (ground truth formation).
[multiChData]←multiChDataForSubject ▹ A subject’s EEG data minLen←0.1 ▹ in second n←30 ▹ amount of channels minFreq←30 ▹ in hertz maxFreq←300 ▹ in hertz lFreq←110 ▹ in hertz hFreq←140 ▹ in hertz [thresholdZScores]←[5,4.9,4.8,…2] ▹ in Z scores (with a -0.1 step) alpha←0.01 ▹ Alpha Value for U-test bestRatio←0 ▹ valid interval over all intervals idealTh←0 ▹ in Z scores $∥\begin{array}{c} EEGSpectrum \end{array}∥\leftarrow getPSDWelch\left(multiChData,minFreq,maxFreq\right)$ [avgSpecFreqAcrossCh]=mean(EEGSpectrum) [powerInData]=[avgSpecFreqAcrossCh]∗∗2 **for** *t* in [thresholdZScores] **do** [intervals]←getMuscleArtIntervals(multiChData,lFreq,hFreq,t,minLen,n) [validIntervals]←[] **for** int in intervals **do** intMultiChData←getMultiChData(int[start],int[len]) $∥\begin{array}{c} EEGSpectrumInt \end{array}∥\leftarrow getPSDWelch\left(intMultiChData,minFreq,maxFreq\right)$ [avgSpecFreqAcrossChInt]=mean(EEGSpectrumInt) [powerInterval]=[avgSpecFreqAcrossChInt]∗∗2 UTest,pValue←mannWhitneyU(powerInData,powerInInt) **if** pValue<alpha **then** validIntervals.append(int) **end if** **end for** ratio←len(validIntervals)/len(intervals) **if** ratio>bestRatio **then** bestRatio←ratio idealTh←t **end if** **end for** [validIntervals]←getMuscleArtIntervals(multiChData,lFreq,hFreq,idealTh,minLen,n)

## References

[1] Chen X., Xu X., Liu A., Lee S., Chen X., Zhang X., McKeown M.J., Wang Z.J. (2019). Removal of muscle artifacts from the EEG: A review and recommendations. IEEE Sens. J..

[2] Nolan H., Whelan R., Reilly R.B. (2010). FASTER: Fully automated statistical thresholding for EEG artifact rejection. J. Neurosci. Methods.

[3] Longo L., Reilly R.B. (2025). onEEGwaveLAD: A fully automated online EEG wavelet-based Learning Adaptive Denoiser for artefacts identification and mitigation. PLoS ONE.

[4] Muthukumaraswamy S.D. (2013). High-frequency brain activity and muscle artifacts in MEG/EEG: A review and recommendations. Front. Hum. Neurosci..

[5] Safieddine D., Kachenoura A., Albera L., Birot G., Karfoul A., Pasnicu A., Biraben A., Wendling F., Senhadji L., Merlet I. (2012). Removal of muscle artifact from EEG data: Comparison between stochastic (ICA and CCA) and deterministic (EMD and wavelet-based) approaches. EURASIP J. Adv. Signal Process..

[6] Barthélemy Q., Mayaud L., Renard Y., Kim D., Kang S.W., Gunkelman J., Congedo M. (2017). Online denoising of eye-blinks in electroencephalography. Neurophysiol. Clin..

[7] McMenamin B.W., Shackman A.J., Maxwell J.S., Bachhuber D.R., Koppenhaver A.M., Greischar L.L., Davidson R.J. (2010). Validation of ICA-based myogenic artifact correction for scalp and source-localized EEG. Neuroimage.

[8] Bhardwaj S., Jadhav P., Adapa B., Acharyya A., Naik G.R. (2015). Online and automated reliable system design to remove blink and muscle artefact in EEG. Proceedings of the 2015 37th Annual International Conference of the IEEE Engineering in Medicine and Biology Society (EMBC).

[9] Nicolaou N., Nasuto S.J. (2007). Automatic artefact removal from event-related potentials via clustering. J. VLSI Signal Process. Syst. Signal Image Video Technol..

[10] Daly I., Scherer R., Billinger M., Müller-Putz G. (2014). FORCe: Fully online and automated artifact removal for brain-computer interfacing. IEEE Trans. Neural Syst. Rehabil. Eng..

[11] Schmoigl-Tonis M., Schranz C., Müller-Putz G.R. (2023). Methods for motion artifact reduction in online brain-computer interface experiments: A systematic review. Front. Hum. Neurosci..

[12] Dora M., Holcman D. (2022). Adaptive single-channel EEG artifact removal with applications to clinical monitoring. IEEE Trans. Neural Syst. Rehabil. Eng..

[13] Donoho D.L., Johnstone I.M. (1998). Minimax estimation via wavelet shrinkage. Ann. Stat..

[14] Donoho D.L., Johnstone I.M. (1994). Threshold selection for wavelet shrinkage of noisy data. Proceedings of the 16th Annual International Conference of the IEEE Engineering in Medicine and Biology Society.

[15] Johnstone I.M., Silverman B.W. (1997). Wavelet threshold estimators for data with correlated noise. J. R. Stat. Soc. Ser. B (Stat. Methodol.).

[16] Bajaj N., Carrión J.R., Bellotti F., Berta R., De Gloria A. (2020). Automatic and tunable algorithm for EEG artifact removal using wavelet decomposition with applications in predictive modeling during auditory tasks. Biomed. Signal Process. Control.

[17] Shin K.G., Ramanathan P. (1994). Real-time computing: A new discipline of computer science and engineering. Proc. IEEE.

[18] Nicolas-Alonso L.F., Gomez-Gil J. (2012). Brain computer interfaces, a review. Sensors.

[19] Kobler R.J., Sburlea A.I., Lopes-Dias C., Schwarz A., Hirata M., Müller-Putz G.R. (2020). Corneo-retinal-dipole and eyelid-related eye artifacts can be corrected offline and online in electroencephalographic and magnetoencephalographic signals. NeuroImage.

[20] Mallat S. (1989). A theory for multiresolution signal decomposition: The wavelet representation. IEEE Trans. Pattern Anal. Mach. Intell..

[21] Mallat S., Zhong S. (1992). Characterization of signals from multiscale edges. IEEE Trans. Pattern Anal. Mach. Intell..

[22] Al-Qazzaz N.K., Hamid Bin Mohd Ali S., Ahmad S.A., Islam M.S., Escudero J. (2015). Selection of mother wavelet functions for multi-channel EEG signal analysis during a working memory task. Sensors.

[23] Gandhi T., Panigrahi B.K., Anand S. (2011). A comparative study of wavelet families for EEG signal classification. Neurocomputing.

[24] Rafiee J., Tse P., Harifi A., Sadeghi M. (2009). A novel technique for selecting mother wavelet function using an intelligent fault diagnosis system. Expert Syst. Appl..

[25] Dragotti P.L., Vetterli M. (2003). Wavelet footprints: Theory, algorithms, and applications. IEEE Trans. Signal Process..

[26] Chen X., Gupta R.S., Gupta L. (2022). Exploiting the Cone of Influence for Improving the Performance of Wavelet Transform-Based Models for ERP/EEG Classification. Brain Sci..

[27] Nobach H., Tropea C., Cordier L., Bonnet J.P., Delville J., Lewalle J., Farge M., Schneider K., Adrian R. (2007). Review of some fundamentals of data processing. Springer Handbooks.

[28] Torrence C., Compo G.P. (1998). A practical guide to wavelet analysis. Bull. Am. Meteorol. Soc..

[29] Lee G., Gommers R., Waselewski F., Wohlfahrt K., O’Leary A. (2019). PyWavelets: A Python package for wavelet analysis. J. Open Source Softw..

[30] Chandola V., Banerjee A., Kumar V. (2009). Anomaly detection: A survey. ACM Comput. Surv. (CSUR).

[31] Liu F.T., Ting K.M., Zhou Z.H. Isolation Forest. Proceedings of the 2008 Eighth IEEE International Conference on Data Mining.

[32] Murphy R.B. (1951). On Tests for Outlying Observations.

[33] Windley P.F. (1960). Trees, forests and rearranging. Comput. J..

[34] Hariri S., Kind M.C., Brunner R.J. (2021). Extended Isolation Forest. IEEE Trans. Knowl. Data Eng..

[35] Young R.K. (2012). Wavelet Theory and Its Applications.

[36] Yochum M., Binczak S. (2015). A wavelet based method for electrical stimulation artifacts removal in electromyogram. Biomed. Signal Process. Control.

[37] Phadikar S., Sinha N., Ghosh R., Ghaderpour E. (2022). Automatic muscle artifacts identification and removal from single-channel eeg using wavelet transform with meta-heuristically optimized non-local means filter. Sensors.

[38] Atangana R., Tchiotsop D., Kenne G., Djoufack L. (2020). Suitable mother wavelet selection for EEG signals analysis: Frequency bands decomposition and discriminative feature selection. Signal Image Process. Int. J..

[39] Endres D.M., Schindelin J.E. (2003). A new metric for probability distributions. IEEE Trans. Inf. Theory.

[40] Fuglede B., Topsoe F. (2004). Jensen-Shannon divergence and Hilbert space embedding. Proceedings of the International Symposium on Information Theory, ISIT 2004.

[41] Luck S.J. (2014). An Introduction to the Event-Related Potential Technique.

[42] Kappenman E.S., Farrens J.L., Zhang W., Stewart A.X., Luck S.J. (2021). ERP CORE: An open resource for human event-related potential research. NeuroImage.

[43] Gasser T., Schuller J.C., Gasser U.S. (2005). Correction of muscle artefacts in the EEG power spectrum. Clin. Neurophysiol..

[44] Kilicarslan A., Grossman R.G., Contreras-Vidal J.L. (2016). A robust adaptive denoising framework for real-time artifact removal in scalp EEG measurements. J. Neural Eng..

[45] Huang J., Wang C., Zhao W., Grau A., Xue X., Zhang F. (2024). LTDNet-EEG: A Lightweight Network of Portable/Wearable Devices for Real-Time EEG Signal Denoising. IEEE Trans. Consum. Electron..

[46] Miyakoshi M. (2023). Artifact subspace reconstruction: A candidate for a dream solution for EEG studies, sleep or awake. Sleep.

[47] Blum S., Jacobsen N.S., Bleichner M.G., Debener S. (2019). A Riemannian modification of artifact subspace reconstruction for EEG artifact handling. Front. Hum. Neurosci..

